# Study of the effect of antimicrobial peptide mimic, CSA-13, on an established biofilm formed by *Pseudomonas aeruginosa*

**DOI:** 10.1002/mbo3.77

**Published:** 2013-02-25

**Authors:** Carole Nagant, Betsey Pitts, Philip S Stewart, Yanshu Feng, Paul B Savage, Jean-Paul Dehaye

**Affiliations:** 1 Laboratoire de Chimie biologique et médicale et de Microbiologie pharmaceutique, Faculté de Pharmacie, Université libre de BruxellesBrussels, Belgium; 2 Center for Biofilm Engineering and Department of Chemical and Biological Engineering, Montana State University-BozemanBozeman, Montana, 59717-3980; 3 Department of Chemistry and Biochemistry, C100 BNSN Brigham Young UniversityProvo, Utah, 84602

**Keywords:** Biofilms, CDC bioreactor, ceragenins, cystic fibrosis.

## Abstract

The formation of a *Pseudomonas aeruginosa* biofilm, a complex structure enclosing bacterial cells in an extracellular polymeric matrix, is responsible for persistent infections in cystic fibrosis patients leading to a high rate of morbidity and mortality. The protective environment created by the tridimensional structure reduces the susceptibility of the bacteria to conventional antibiotherapy. Cationic steroid antibiotics (CSA)-13, a nonpeptide mimic of antimicrobial peptides with antibacterial activity on planktonic cultures, was evaluated for its ability to interact with sessile cells. Using confocal laser scanning microscopy, we demonstrated that the drug damaged bacteria within an established biofilm showing that penetration did not limit the activity of this antimicrobial agent against a biofilm. When biofilms were grown during exposure to shear forces and to a continuous medium flow allowing the development of robust structures with a complex architecture, CSA-13 reached the bacteria entrapped in the biofilm within 30 min. The permeabilizing effect of CSA-13 could be associated with the death of the bacteria. In static conditions, the compound did not perturb the architecture of the biofilm. This study confirms the potential of CSA-13 as a new strategy to combat persistent infections involving biofilms formed by *P. aeruginosa*.

## Introduction

Cystic fibrosis is the most common recessively inherited exocrinopathy in Caucasians affecting 1 in 3000 newborns (Kosorok et al. 1996). It is provoked by mutations of the cystic fibrosis transmembrane regulator (CFTR), a protein involved in chloride and sodium movements in exocrine glands (Muallem and Vergani 2009). In the upper airways, the secretions of these patients are extremely viscous and difficult to eliminate leading to clinical symptoms like chronic cough, wheezing, sputum production, air trapping, and digital clubbing and to radiological images of pulmonary atelectasis, bronchiectasis, hyperinflation, infiltrations, and nasal polyps (Loeve et al. 2011). Pulmonary infections are the major cause of deaths among patients with cystic fibrosis (Lyczak et al. 2002). Extensive use of new modes of delivery of antimicrobial and mucolytic drugs combined with intensive physical therapy has contributed to the improvement of the pulmonary symptoms of these patients and to the increase of their median survival age which now exceeds 30 years (Dodge et al. 2007). Longitudinal studies of these infections reveal that the pathogens vary during the lifetime of the patients (Gilligan 1991). The upper airways of CF children from infancy to mid childhood are mostly infected by Gram-positive bacteria, especially *Staphylococcus aureus*. The tendency changes with CF young adults who are colonized mostly by Gram-negative bacteria; 80% of the patients are eventually colonized by *Pseudomonas aeruginosa* (Hauser et al. 2011). Recurrent infections involving *P. aeruginosa* eventually lead to chronic infections which worsen the prognosis and finally provoke the death of the patients. There has been no clear explanation for the predisposition of CF patients to infections by *P. aeruginosa* (Davies and Bilton 2009) but the correlation between colonization of upper airways by these bacteria and poor outcome of the disease has been clearly demonstrated (McPhail et al. 2008). The transition from an acute to a chronic infection is secondary to mutations provoking major alterations of the phenotype of *P. aeruginosa* (Rodríguez-Rojas et al. 2012). They become mucoid, secreting alginate which forms an extracellular matrix contributing to the aggregation between cells or to their adhesion on surfaces, the first steps in the development of a biofilm (Hassett et al. 2010). Within the biofilm, some bacteria are much less sensitive to treatment (Stewart and Costerton 2001) and patients become chronically infected. Moreover, the emergence of multidrug resistant bacteria is a concern for the use of conventional antibiotherapy (Talbot et al. 2006). In order to circumvent this problem, alternatives to classical antibiotics are extensively studied.

**Figure 1:**
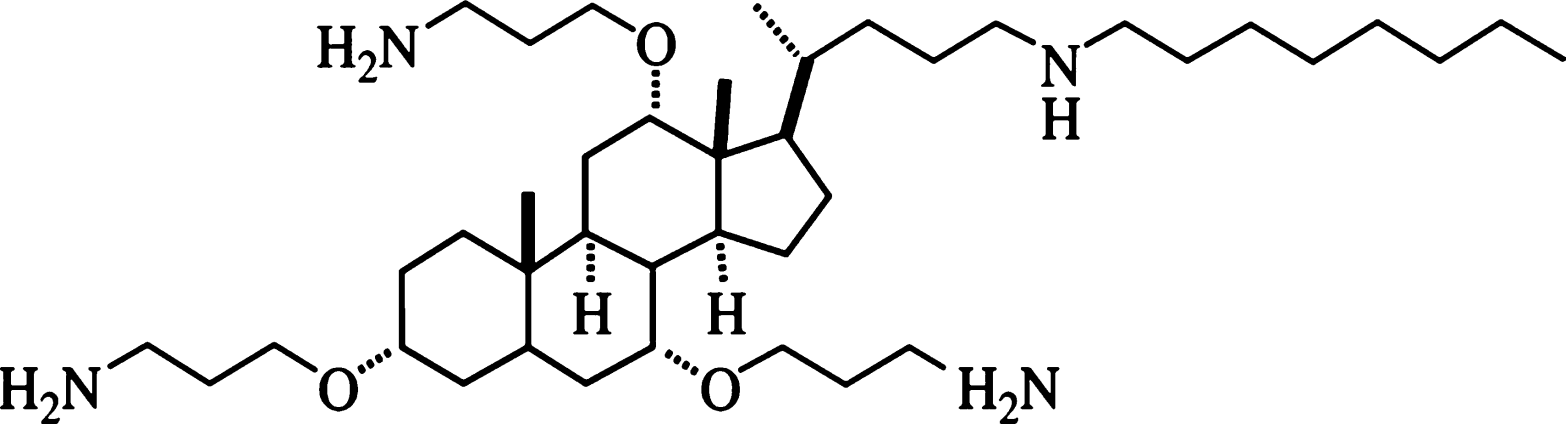
Structure of ceragenin cationic steroid antibiotics (CSA)-13.

Antimicrobial peptides are cationic peptides secreted by leukocytes or epithelial cells and which form amphipathic alpha helices or short beta sheets (Seil et al. 2010). Recent data demonstrate that LL-37, the human peptide derived from cathelicidin, is active against *P. aeruginosa* not only in planktonic cultures but also within biofilms (Nagant et al. 2012). Antimicrobial peptides are sensitive to the proteases expressed and secreted by host cells as well as by *P. aeruginosa* which limits the prospect of their therapeutic use (Moncla et al. 2011). In order to circumvent this issue, synthetic analogs of the antimicrobial peptides have been developed. Ceragenins are a promising class of synthetically produced molecules derived from cholic acid, a common bile acid to which aminoalkyl groups have been added to the alcohol groups (Li et al. 1998). The amino groups are protonated at neutral pH hence the name of cationic steroid antibiotics (CSA) also given to these molecules (Epand et al. 2008). As the hydroxyl groups of bile acids are oriented on one face of the molecule, these compounds are facially amphiphiles with one hydrophobic side formed by the sterane ring and the positive charges of the amino groups forming the hydrophilic face of the molecule. In this way, ceragenins share the cationic and amphiphatic properties of antimicrobial peptides and it has been postulated that these agents share a similar mechanism of action by targeting the bacterial membrane (Ding et al. 2002). Previous results have demonstrated the efficacy of CSA-13 (Fig. 1), a lead ceragenin, against *P. aeruginosa* not only in planktonic cultures but also within biofilms (Nagant et al. 2010). This drug also prevents the formation of a biofilm on a polystyrene surface (Nagant et al. 2011). In the present study, we showed that the drug was able to affect the bacteria not only at the surface but also inside the biofilm showing that penetration does not limit the activity of this antimicrobial agent against a biofilm. CSA-13 did not destroy the architecture of the biofilm under our experimental conditions.

## Materials and Methods

### Strains and growth condition

The *P. aeruginosa* reference strains ATCC 15692 PA01 and ATCC 15442 were stored at -80°C in skimmed milk. The *P. aeruginosa* strains expressing the green fluorescent protein (GFP), PAO1 (pMF230) (Nivens et al. 2001), and the red fluorescent protein (RFP), PAO1 (pMF440) (unpublished), were kindly provided by Pr M. Franklin (Center for Biofilm Engineering, Bozeman, MT). Both plasmids contain a carbenicillin resistance marker. Before use, bacterial colonies were spread onto tryptic soy agar (TSA) medium and incubated at 37°C for 24 h. The bacteria were transferred from plate to plate not more than three times onto TSA medium.

### Study of the effect of CSA-13 on biofilms formed in a microtiter plate

An overnight growth culture of PAO1 in tryptic soy broth (TSB) was adjusted to a final O.D._600 nm_ of 0.01. Two hundred and fifty microliters of the bacterial suspension was added to the wells of a polystyrene 96-well microtiter plate with a μClear base (Greiner Bio-One, France). The plate was incubated at 37°C on an orbital shaker (30 rpm). The medium was changed after 3 h to remove non attached bacteria. After 24-h incubation, the bacteria were rinsed and exposed to increasing concentrations of CSA-13 (0, 20, 50, and 100 mg/L). After 1 h, the wells were rinsed and stained for 25 min with the BacLight™ Live/Dead® staining kit (Molecular Probes, Eugene, OR) (final concentrations of Syto9 and propidium iodide (PI): 30 μmol/L and 120 μmol/L, respectively). After rinsing, the wells were examined by confocal laser scanning microscopy (CLSM) with a Leica SP5 (Leica Microsystems Inc., Buffalo Grove, IL) using a 63× objective with a 1.2 numerical aperture. Excitation was performed using two lasers at 488 and 561 nm and the fluorescence emission was collected from 500 to 550 nm and from 570 to 700 nm. Images were scanned at 600 Hz and analyzed with the Imaris software (Bitplane, Zurich, Switzerland).

An overnight planktonic bacterial culture of *P. aeruginosa* PAO1-GFP in TSB supplemented with 150 mg/L carbenicillin (Fisher BioReagents, Fair Lawn, NJ), to maintain the GFP plasmid (pMF230), was added to the wells of a microtiter plate and cells were incubated in the conditions previously described. After 24-h incubation, the wells were rinsed and mounted on the stage of the confocal microscope. CSA-13 (final concentration 100 mg/L) and PI (final concentration 60 μmol/L) were added to the wells and series of images were taken during 30 min. A control was performed in parallel in the presence of PI and in the absence of CSA-13.

### Study of the effect of CSA-13 on biofilms formed in a “Center for Disease Control” biofilm reactor

*Pseudomonas aeruginosa* PAO1-GFP-tagged biofilms were grown in center for disease control (CDC) biofilm reactors (Biosurface Technologies Inc., Bozeman, MT). This method is routinely used to study the formation of biofilms by *P. aeruginosa* grown with high shear and continuous flow (Goeres et al. 2005). It consists of growing biofilms on glass coupons held in rods immersed in a glass vessel. A continuous flow provides nutrient medium at a constant flow rate and a shear is generated by a magnetic stirring. Briefly, a few colonies were grown overnight in 100 mL of 1:100 strength TSB (300 mg/L) with 150 mg/L carbenicillin, to maintain the GFP plasmid (pMF230), at 37°C under constant shaking (120 rpm). The next day, 1 mL of the broth was transferred to the reactor containing 500 mL TSB (300 mg/L) with 150 mg/L carbenicillin. The reactor was incubated for 24 h at room temperature with constant stirring at 120 rpm. The reactor was then connected to a nutrient carboy containing 1:300 strength TSB (100 mg/L) to allow a continuous flow at a 12 mL/min rate for 24 h. Biofilms grown on the glass coupons were removed from the reactor, rinsed with distilled water, and exposed to CSA-13 and 60 μmol/L PI for 25 min. After contact, the biofilms were rinsed, mounted on the stage of the confocal microscope, and observed using a 63× water immersion objective with a 0.9 numerical aperture. Excitation was performed using two lasers at 488 and 561 nm and the fluorescence emission was collected from 500 to 550 nm and from 570 to 700 nm. Images were scanned at 600 Hz and analyzed with the Imaris software. Similar experiments were performed with the reference strain ATCC 15442. After the formation of the biofilm, the coupons were delicately removed and exposed for 25 min to CSA-13 (0, 100, or 200 mg/L). The coupons were then rinsed and stained with 100 μL of the BacLight™ Live/Dead® staining kit (final concentrations of Syto9 and PI of 15 μmol/L and 60 μmol/L, respectively). After rinsing, the coupons were examined as previously described.

### Study of the interaction of labeled CSA-13 with biofilms formed in a CDC biofilm reactor

These studies were performed with the PAO1-RFP strain. The CSA-13 was labeled with 1% bodipy as previously described (Nagant et al. 2011). After formation of the biofilm by the PAO1-RFP strain, the coupons were delicately removed, rinsed, and incubated with fluorescent CSA-13 (100 and 200 mg/L) for 25 min. The coupons were examined as previously described using CLSM.

## Results

### Studies on biofilms formed on the bottom of the wells of a microtiter plate

**Figure 2:**
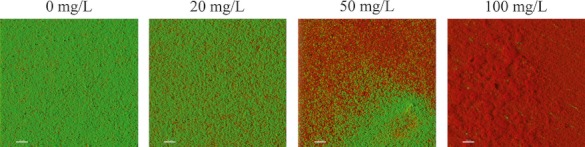
Effect of various concentrations of cationic steroid antibiotics (CSA)-13 on a biofilm preformed by PAO1 in the wells of a microtiter plate. Biofilms were formed in the wells of a microtiter plate for 24 h by the PAO1 strain and incubated in the presence of 0, 20, 50, and 100 mg/L CSA-13 for 25 min. After treatment, cells were stained using the BacLight™ Live/Dead® staining kit. Healthy cells are green and damaged cells are red. These images are representative of two experiments. Size bars correspond to 20 μm.

A PAO1 biofilm was formed for 24 h in the wells of a microtiter plate. After treatment, the cells were stained with the BacLight™ Live/Dead® staining kit and examined by CLSM. As shown in Figure 2, the cells formed a uniform and smooth biofilm. All the cells of the field were stained with Syto9 and appeared green. The lack of staining with PI confirmed the viability of the bacteria within the biofilm in our experimental conditions. Increasing concentrations of CSA-13 were added to adjacent wells. At 20 mg/L, the drug already provoked the appearance of damaged cells (small red spots) homogeneously dispersed in the biofilm. At 50 mg/L, a large area of the surface of the biofilm was red showing that a vast majority of the cells had become unable to exclude PI. At 100 mg/L CSA-13, all the cells were permeant to the dye and the entire surface of the biofilm had turned red. It could be concluded that CSA-13 was active against bacteria forming a thin biofilm.

**Figure 3:**
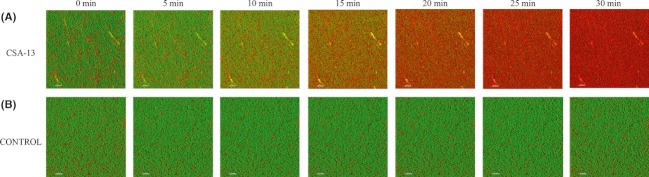
Time course of the effect of CSA-13 on a biofilm preformed by PAO1-GFP in the wells of a microtiter plate. Biofilms were formed in the wells of a microtiter plate for 24 h by the PAO1-GFP strain and incubated in control conditions (lower part) or in the presence of 100 mg/L CSA-13 (upper panel). PI (60 μmol/L) was added to the wells. The wells were photographed every 5 min for 30 min. Healthy cells are green and damaged cells are red. These images are representative of two experiments. Size bars correspond to 20 μm. CSA, cationic steroid antibiotics; GFP, green fluorescent protein; PI, propidium iodide.

In the next experiment, the effect of 100 mg/L CSA-13 was tested at various times. As shown in Figure 3, the antimicrobial effect of CSA-13 was very rapid: after 10 min, a significant increase of the uptake of PI could already be observed. After 20 min, all the bacteria were killed and the entire surface of the biofilm had turned red.

### Studies on biofilms formed on coupons of a CDC bioreactor

**Figure 4:**
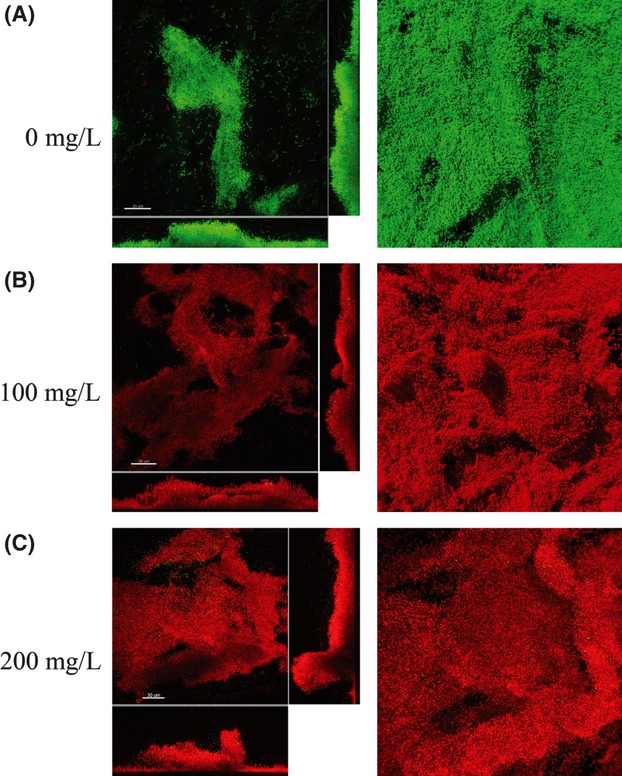
Effect of CSA-13 on a biofilm preformed by PAO1-GFP in a CDC bioreactor. The PAO1-GFP biofilm was formed for 24 h in a CDC bioreactor. The biofilm was then exposed for 25 min to 0, 100, or 200 mg/L CSA-13 in the presence of 60 μmol/L PI. Left column: The central images show a view in the *xy* plan and the flanking bottom and right pictures show side views of the biofilm in the *xz* and *yz* plans, respectively. Right column: Reconstituted tridimensional image of a top view of the biofilm. The size bars correspond to 30 μm. CSA, cationic steroid antibiotics; GFP, green fluorescent protein; CDC, center for disease control; PI, propidium iodide.

In the previous experiments, biofilms were formed under static conditions which results in rather flat biofilms. In order to produce a more structured biofilm, the next experiments were performed on biofilms formed under fluid shear stress. The coupons of a CDC bioreactor were constantly perfused for 24 h before being exposed to the tested drug in static conditions and examined with CLSM. As shown in Figure 4A, the surface of the biofilm was irregular and its architecture much more complex. Bacteria expressing GFP could be observed not only on the surface of the coupon but also in columnar vertical extensions giving the biofilm a tridimensional architecture. After a short exposure, the drug affected the viability of the bacteria at any location in the biofilm. Bacteria located at the surface of the biofilm or cells deeply hidden at the basis of a columnar upward extension were equally affected by CSA-13. These results showed that the extracellular matrix of the biofilm did not block the penetration of the drug to the depth of the biofilm. When exposed to 100 mg/L CSA-13, the structure of the biofilm was not affected and the upward extensions of bacterial colonies could still be observed. Increasing the concentration of CSA-13 to 200 mg/L did not modify the results: the drug increased the permeability of the bacteria to PI without modifying the tridimensional architecture of the biofilm. These results suggested that CSA-13 was bactericidal on bacteria trapped in a complex biofilm but after a short exposure in static conditions it could not eradicate the biofilm itself.

**Figure 5:**
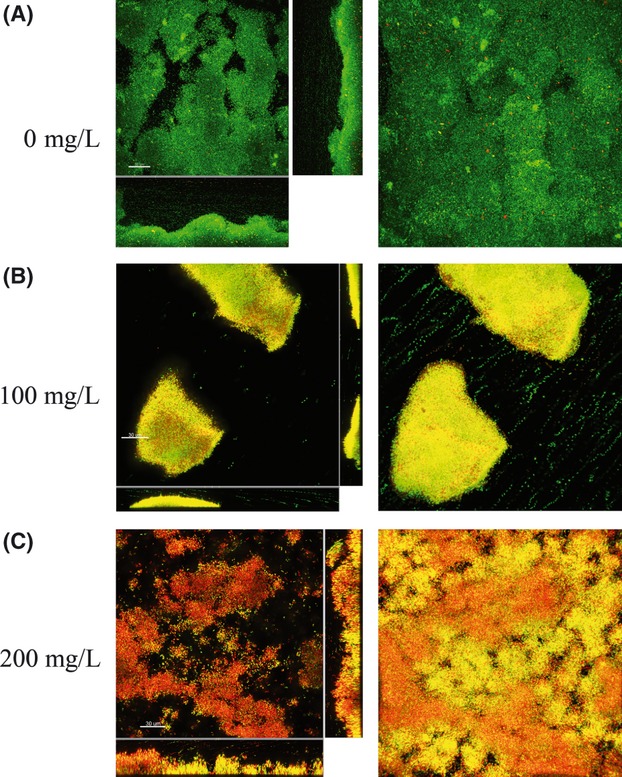
Effect of CSA-13 on a biofilm preformed by ATCC 15442 in a CDC bioreactor. The biofilm was grown for 24 h by the ATCC 15442 reference strain in a CDC bioreactor. The biofilm was then exposed for 25 min in the presence of 0, 100 mg/L, or 200 mg/L CSA-13. After rinsing, the biofilm was stained with the BacLight™ Live/Dead® staining kit. Healthy cells appear green and damaged cells appear red. Left column: The central images show a view in the *xy* plan and the flanking bottom and right pictures show side views of the biofilm in the *xz* and *yz* plans, respectively. Right column: Reconstituted tridimensional image of a top view of the biofilm. The size bars correspond to 30 μm. CSA, cationic steroid antibiotics; CDC, center for disease control.

The experiment was repeated with ATCC 15442, another reference strain of *P. aeruginosa*. The BacLight™ Live/Dead® staining kit was used to stain living cells with Syto9 (green fluorescence) and to stain dead cells with PI (red fluorescence) (Fig. 5). At 100 mg/L, CSA-13 affected the integrity of most cells of the microcolonies located at the surface of the biofilm (Fig. 5B, left figure) but cells located in some parts of the bottom of the biofilm remained green and were not affected by 100 mg/L CSA-13. Doubling the concentration of CSA-13 affected the integrity of the cells in the entire biofilm (Fig. 5C).

**Figure 6:**
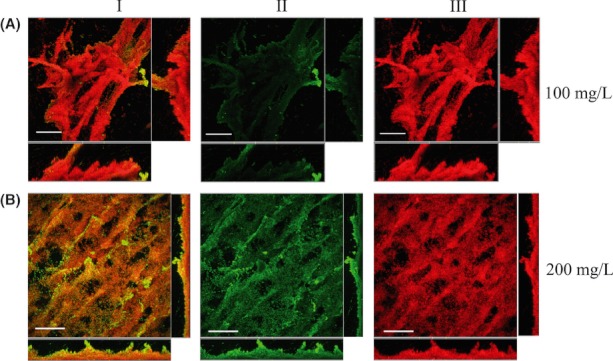
Interaction of bodipy labeled CSA-13 with bacteria in a biofilm preformed by the PAO1-RFP strain in a CDC bioreactor. A biofilm was grown for 24 h by the PAO1-RFP strain in a CDC bioreactor. The biofilm was then exposed for 25 min to 100 mg/L or 200 mg/L bodipy labeled CSA-13. Cells express the red fluorescent protein and appear red; cells which bind the bodipy CSA-13 appear green. Left column (I): The central images show a view in the *xy* plan and the flanking bottom and right pictures show side views of the biofilm in the *xz* and *yz* plans, respectively. Middle and right columns (II and III): Deconvolution of the left image for the green (bodipy CSA-13) and red (red fluorescent protein) dyes. Size bars correspond to 50 μm. CSA, cationic steroid antibiotics; RFP, red fluorescent protein; CDC, center for disease control.

In the last experiment, the access of a fluorescent analog of CSA-13 to the interior of the biofilm was examined using bodipy CSA-13, a fluorescent analog of the drug. As bodipy and GFP have comparable spectra, these experiments were performed with RFP-tagged bacteria rather than with PAO1-GFP. As shown in Figure 6, the fluorescent drug (100 mg/L and 200 mg/L) stained all the cells of the biofilm confirming that the extracellular matrix did not limit its access to the inner part of the biofilm.

## Discussion

Using two different methods to develop a biofilm, we clearly established that CSA-13, a mimic of endogenous antimicrobial peptides, was active against bacteria inside a biofilm. Our results also show that the drug, after a short exposure in static conditions, did not modify the structure of the biofilm.

Previous results have shown that CSA-13 inhibited the adhesion of *P. aeruginosa* on an abiotic surface (Nagant et al. 2011) and also that it was bactericidal on cells within a mature biofilm formed on the pegs of a lid of a microtiter plate (Nagant et al. 2010). The activity on sessile cells was confirmed using CLSM. When exposed for a very short period to CSA-13, the bacteria trapped in a biofilm formed at the bottom of a microtiter plate were permeable to PI and turned red. These cells were dead as confirmed by enumeration (Nagant et al. 2010). Microscopic images also showed that the biofilms formed in static conditions on the bottom of wells had a very flat architecture and did not reproduce the geometry of in vivo biofilms. Biofilms formed under constant shear stress (magnetic stirring of the medium, perfusion of the culture medium) are more complex and develop not only on the surface but also via vertical extensions. Biofilms grown in these conditions using a CDC bioreactor were very different from the biofilms formed at the bottom of a well and had a much more complex and robust structure. The biofilms formed in the CDC bioreactor have been extensively studied and have been validated as a good model for in vivo biofilms (Hadi et al. 2010; American Society for Testing and Materials 2012). CSA-13 was as effective on these biofilms as on flat biofilms. This result suggested that the complex extracellular matrix did not interfere with the antimicrobial activity of the drug which had full access to all the bacteria inside the biofilm in agreement with other measurements of adequate penetration of antibiotics into biofilms (Davison et al. 2010). This conclusion was comforted with the fluorescent analog of CSA-13 which interacted with the bacteria throughout the biofilm. Higher concentrations of CSA-13 were needed to observe the same effect on the entire ATCC 15442 biofilm. This is consistent with previous results showing that this strain was less sensitive to CSA-13 than the PAO1 strain (Nagant et al. 2010). As previously reported (Nagant et al. 2010), concentrations of CSA-13 active on sessile cells were much higher than those active on planktonic cultures (MIC 6–12 mg/L for PAO1 and 3 mg/L for ATCC 15442). Our results agree with previous results of Pollard et al. (2009). Using colony counting, they studied the effect of CSA-13 on established biofilms formed by *P. aeruginosa* in the CDC bioreactor. After a long-term (72 h) exposure, the drug proved to be bactericidal with an efficacy better than that of ciprofloxacin. The study of the biofilms with CLSM made it possible to demonstrate that CSA-13 was anti-microbial on all the cells entrapped in a biofilm and that it did not modify the structure of the biofilm after a short exposure in static conditions. This is reminiscent of the results of Hadi et al. (2010) who showed that a biofilm formed in a CDC bioreactor was differently affected by sodium hydroxide which killed bacteria and destroyed the biofilm and Tween20 which killed the bacteria without affecting the biofilm. It might be expected that the damaged cells within the biofilm contribute to the destabilization of the global biofilm structure and that in vivo conditions (e.g., mucociliary clearance) help to completely destroy the already weakened biofilm. Destruction of the biofilm by a drug like N-acetylcysteine (Zhao and Liu 2010) or DNAse (Fuxman Bass et al. 2010) is secondary to the effect on the matrix, not on the bacteria. Considering that biofilm removal and bacterial killing are distinct processes (Chen and Stewart 2000), a concomitant administration of CSA-13 with factors affecting the biofilm matrix might be needed to completely dismantle and eradicate the biofilm. Nevertheless, our results demonstrate that CSA-13 effectively kills well ensconced cells within established biofilms.

Though failure of antibiotics to penetrate a biofilm has often been advanced as an explanation for biofilm resistance to antimicrobial chemotherapy, direct measurements of antibiotic penetration do not support this hypothesis (Stone et al. 2002; Zheng and Stewart 2002; Walters et al. 2003). Neither is CSA-13 hindered in its access to the interior of a *P. aeruginosa* biofilm. Considering its permeabilizing properties on the bacterial membrane, its resistance to degradation, its preventive effect on bacterial adhesion on surfaces, and its ability to diffuse within a biofilm, CSA-13 is thus a potential candidate to treat not only acute infections caused by planktonic bacteria but also chronic infections involving biofilms.
